# Pretreatment CT differential diagnosis of tuberculous peritonitis from peritoneal carcinomatosis of advanced epithelial ovarian cancer

**DOI:** 10.1038/s41598-023-27771-5

**Published:** 2023-01-19

**Authors:** Chul-min Lee, Joong Sub Choi, Mimi Kim, Bo-Kyeong Kang, Jaeman Bae, Won Moo Lee, Un Suk Jung, Jeong Min Eom, Yeon Kyoung Kim, Jin Young Kim

**Affiliations:** 1 Department of Radiology, Hanyang University College of Medicine, Seoul, Republic of Korea; 2 Division of Gynecology and Gynecologic Minimally Invasive Surgery, Department of Obstetrics and Gynecology, Hanyang University College of Medicine, 222 Wangsimni-ro Seongdong-gu, Seoul, 04763 Republic of Korea

**Keywords:** Cancer, Microbiology, Diseases

## Abstract

Delayed diagnosis of female genital tuberculosis (FGTB) can lead to inappropriate treatment and unnecessary surgical procedures rather than standard anti-TB medication. We tried to evaluate the use of computed tomography (CT) imaging to differentiate TB peritonitis from peritoneal carcinomatosis of advanced epithelial ovarian cancer (AEOC). We investigated women who underwent CT to distinguish between TB peritonitis and peritoneal carcinomatosis of AEOC. We evaluated various CT imaging features to identify differences between the two diseases. In addition, we performed univariate and multivariate logistic regression analyses to identify the independent imaging parameters associated with TB peritonitis and evaluated the diagnostic performance of the combined imaging parameters. We also performed the histopathological analysis of the available salpinx specimens of TB peritonitis. We included 25 women with TB peritonitis and 34 women with peritoneal carcinomatosis of AEOC. A multivariate analysis of the discriminant CT imaging features between the two diseases revealed that changes in fallopian tubes and peritoneal micronodules were independent parameters associated with TB peritonitis (*p* ≤ 0.012). Combining the two imaging parameters showed an area under the receiver operating characteristic curve of 0.855, a sensitivity of 88.0%, and a specificity of 67.7% for differentiating TB peritonitis from peritoneal carcinomatosis. Furthermore, changes in fallopian tubes were correlated with histopathological abnormalities in salpinx specimens. Pretreatment CT evaluation with useful imaging features could help differentiate TB peritonitis from peritoneal carcinomatosis of AEOC.

## Introduction

Tuberculosis (TB) is an infectious disease and its eradication is a global priority for the World Health Organization^1^. TB’s extrapulmonary forms are lymph nodal, pleural, and urogenital. Female genital tuberculosis (FGTB) is the fourth most common cause of extrapulmonary TB, which generally affects 20- to 40-year-old women and is rare in postmenopausal women^2,3^. FGTB infection occurs via two mechanisms: that is hematogenous/lymphatic spread from a primary site or sexual transmission^4^. The specific sites of involvement are the fallopian tube (95–100%), endometrium (50–60%), ovary (20–30%), cervix (5–15%), myometrium (2–5%), and vagina/vulva (1%)^5^. FGTB presents with the symptoms of infertility, pelvic pain, vaginal bleeding, amenorrhea, leukorrhea, or postmenopausal bleeding, but is frequently asymptomatic. Physicians often underestimate FGTB because of its rarity and nonspecific symptoms^6^. The delayed diagnosis of FGTB by physicians can lead to inappropriate treatment and notably unnecessary surgical procedures instead of standard anti-TB medication^7^.

FGTB usually accompanies TB peritonitis namely, an extrapulmonary TB with the diffuse involvement of the peritoneal cavity^8^. Computed tomography (CT) is a useful and popular diagnostic imaging modality for evaluating women with abdominal distension. CT scans can identify omental infiltration, peritoneal nodules, and ascites in women with TB peritonitis^9^. These are diagnostic differential findings for women with TB peritonitis and peritoneal carcinomatosis of advanced epithelial ovarian cancer (AEOC) based on the size and shape of the adnexal masses and peritoneal nodules, but it is challenging for gynecologic oncologists and radiologists to find differences between the two diseases^10,11^. Furthermore, it is more difficult to differentiate women without definite ovarian masses in AEOC and women with TB peritonitis in CT scans^12^. Thus a substantial number of women with TB peritonitis undergo laparoscopy because of difficulties in differential diagnosis. Unfortunately, some women may undergo radical and unnecessary surgery, such as unilateral or bilateral salpingo-oophorectomy and hysterectomy, because of the surgeon’s lack of medical proficiency, confused laparoscopic findings related to TB peritonitis and peritoneal carcinomatosis, and inaccessibility of frozen section analysis. These surgeries damage fertility, and affected women may encounter surgical complications throughout their lives. The relationship between patients and their physicians can also deteriorate, leading to medicolegal issues^3,7^. However, few studies have reported differential diagnostic findings for the two diseases based on CT scans^12–15^.

Therefore, our study evaluated clinically whether pretreatment CT imaging features are useful for the differential diagnosis of TB peritonitis from peritoneal carcinomatosis of AEOC.

## Materials and methods

### Study population

The Institutional Review Board of Hanyang University Hospital approved this retrospective study and the requirement for informed consent was waived due to its retrospective nature (IRB No. HYUH 2021-04-089). All methods followed the relevant guidelines and regulations. We conducted a retrospective review of the clinical charts, including the patient’s age, symptoms and signs, surgical procedures, serum levels of tumor markers of CA125 and CA19-9, histopathological reports, surgically related complications, and picture archiving and communication system of women who underwent CT for the differential diagnosis of TB peritonitis and peritoneal carcinomatosis of AEOC from May 2004 to June 2020.

**Figure 1:**
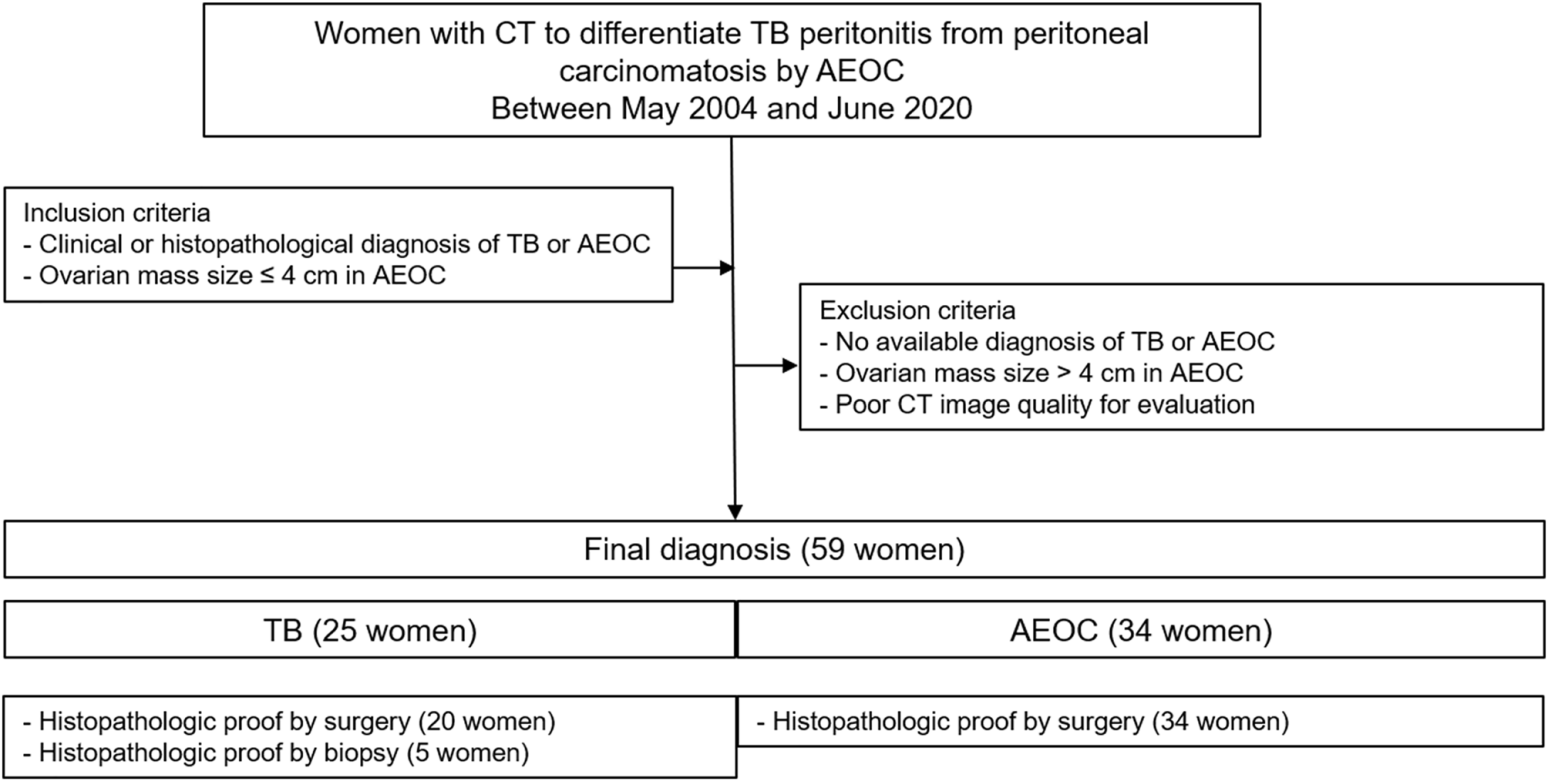
Study population.

The inclusion criteria were as follows: (1) histopathological confirmation of TB peritonitis or peritoneal carcinomatosis of AEOC, (2) available pretreatment CT, and (3) ovarian mass size < 4 cm for peritoneal carcinomatosis. Exclusion criteria were as follows: (1) no available pathological diagnosis, (2) ovarian mass size ≥ 4 cm for peritoneal carcinomatosis because women with TB peritonitis usually have small adnexal mass (< 4 cm), and (3) poor CT image quality for evaluation (Fig. 1). We excluded ovarian cancer patients with an ovarian mass ≥ 4 cm, because the differential diagnosis of TB peritonitis from peritoneal carcinomatosis of normal-sized ovarian cancer is more challenging when the ovarian mass is < 4 cm^12^. Diffuse peritoneal abnormalities accompanied by an adnexal mass large enough to distinguish enhancing solid portion (≥ 4 cm) could be easily diagnosed as peritoneal carcinomatosis by ovarian cancer.

### CT techniques

All women underwent CT examination using 16- or 64-detector row CTs (Somatom Sensation 16, Somatom Definition Edge, and Somatom Definition FLASH [Siemens Healthineers, Erlangen, Germany]; Brilliance 64 [GE Healthcare, Chicago, IL, USA]). An unenhanced image of the pelvis was acquired first, and the portal venous phase of the abdomen and pelvis (PVP; 70 s) was obtained after an intravenous iodine contrast injection of 120–150 mL iodine contrast (Bonorex, Central Medical Service) at a rate of 3 mL/s using a power injector.

### Imaging analysis

Two board-certified radiologists (each with 6 years of experience in genitourinary imaging) independently evaluated the CT images blinded to all women's final diagnoses. Discordant cases were resolved by a third board-certified radiologist with 10 years of experience in genitourinary imaging (anonymized). Initially, the ovarian masses were assessed as none, unilateral, or bilateral. We measured the size of the ovarian mass on the axial scan, and used the mean size in the case of bilateral ovarian masses. The radiologists evaluated the following CT imaging features: (1) presence of changes in fallopian tubes (hydrosalpinx or tubal enhancement); (2) mean ovarian attenuation (mean Hounsfield unit of each ovarian measurement); (3) peritoneal nodule (micronodules or macronodules); (4) pattern of omentum (none, infiltration, nodular, or caked appearance); (5) amount of ascites; and (6) mean ascites attenuation (mean value of two site measurements in the pelvic cavity).

**Figure 2:**
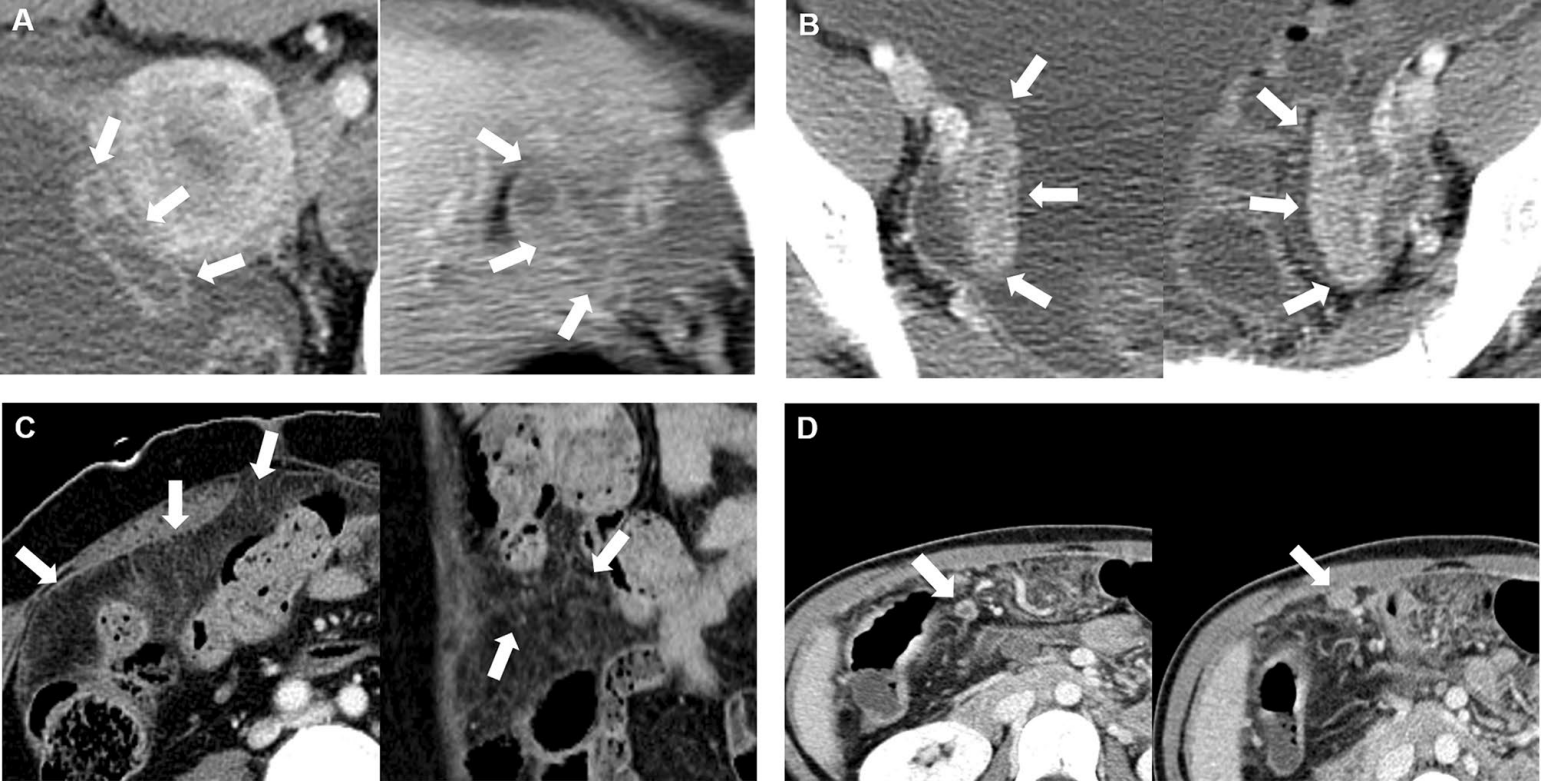
Representative cases of CT imaging features. (**A**) Hydrosalpinx, (**B**) fallopian tube enhancement, (**C**) peritoneal micronodule, and (**D**) peritoneal macronodule.

We defined the peritoneal macronodules when there were any peritoneal nodules or masses the size of ≥ 5 mm. In contrast, the remaining cases showed diffuse peritoneal infiltration and nodularity without discrete peritoneal nodules or masses ≥ 5 mm as peritoneal micronodules. Representative cases for each imaging feature are shown in Fig. 2.

### Reference standard and histopathologic analysis

All 34 women with peritoneal carcinomatosis of AEOC were pathologically confirmed after surgery. Among 25 women with TB peritonitis, 20 (20/25, 80.0%) were pathologically confirmed after surgery. The remaining five women (5/25, 20.0%) were diagnosed with TB peritonitis after percutaneous peritoneal biopsy or ascites aspiration.

We performed the histopathological analysis of five TB peritonitis adnexectomy specimens compared to peritoneal carcinomatosis. Adnexectomy is not usually performed in women with TB peritonitis. However, in the case of these five patients, the uncertainty of surgical findings and frozen section analysis led to adnexectomy, and we could perform histopathologic analysis of TB peritonitis salpinges.

### Statistical analysis

Continuous variables were compared using the Mann–Whitney U test, while categorical variables were compared using the chi-square test between TB peritonitis and peritoneal carcinomatosis. We performed a univariate logistic regression analysis using significantly different parameters between the two diseases. A subsequent multivariate logistic regression analysis was performed to identify independent parameters associated with TB peritonitis. The diagnostic performance of each imaging parameter and their combination for the differential diagnosis of TB peritonitis from peritoneal carcinomatosis was evaluated using the area under the receiver operating characteristic curve (AUC), calculating the sensitivity, specificity, positive and negative predictive values, and diagnostic accuracy. The interobserver agreements for the key imaging features were evaluated between the two radiologists using kappa statistics as follows: 0.00–0.20, poor; 0.21–0.40, fair; 0.41–0.60, moderate; 0.61–0.80, good; and 0.81–1.00, excellent agreement. Statistical significance was set at *p* < *0.05*. All statistical analyses were performed using SPSS software (version 26.0; IBM SPSS, Armonk, NY, USA).

### Ethics approval

The Institutional Review Board of Hanyang University Hospital approved this study.

## Results

### Baseline demographics and clinical characteristics of the patients

In this study, 25 women had TB peritonitis, and 34 had peritoneal carcinomatosis of AEOC. The median age was 57 years (24–96 years) for TB peritonitis and 62 years (33–78 years) for peritoneal carcinomatosis. In TB peritonitis, 13 (52.0%) and five (20.0%) out of 25 women showed bilateral and unilateral ovarian masses, respectively. In CT scans, seven out of 25 women with TB peritonitis (28.0%) showed no discernible ovarian masses. Twenty-nine out of 34 women with peritoneal carcinomatosis (85.3%) showed bilateral ovarian masses, and the remaining five (14.7%) had a unilateral ovarian mass; on the right in one patient and on the left in four patients (Table 1). The median CA125 and CA19-9 levels were not significantly different between the TB peritonitis and peritoneal carcinomatosis (CA125, 352.1 IU/mL vs. 981.1 IU/mL, *p* = 0.052; CA19-9, 5.0 IU/mL vs. 6.7 IU/mL, *p* = 0.470).

**Table 1:** Baseline demographics and clinical characteristics of the patients.

	TB peritonitis (n = 25)	Peritoneal carcinomatosis (n = 34)
Age*	57 (24–96)	62 (33–78)
Abdominal distension		
Yes	24 (96.0)	33 (97.1)
No	1 (4.0)	1 (2.9)
Ovary mass size (cm)*	3.8 (0.0–6.8)	3.1 (1.4–4.0)
Laterality		
No mass	7 (28.0)	0
Unilateral	5 (20.0)	5 (14.7)
Bilateral	13 (52.0)	29 (85.3)
Diagnosis		
Surgery	20 (80.0)	34 (100)
Biopsy	5 (20.0)	0
FIGO stage		
IA		2 (5.9)
IIB		1 (2.9)
IIIA		1 (2.9)
IIIB		6 (17.6)
IIIC		19 (55.9)
IVA		5 (14.7)

Data are the number (percentage) of patients.

*Data are presented as the median (range) of the variables.

### Discriminant CT imaging features between TB peritonitis and peritoneal carcinomatosis

**Figure 3:**
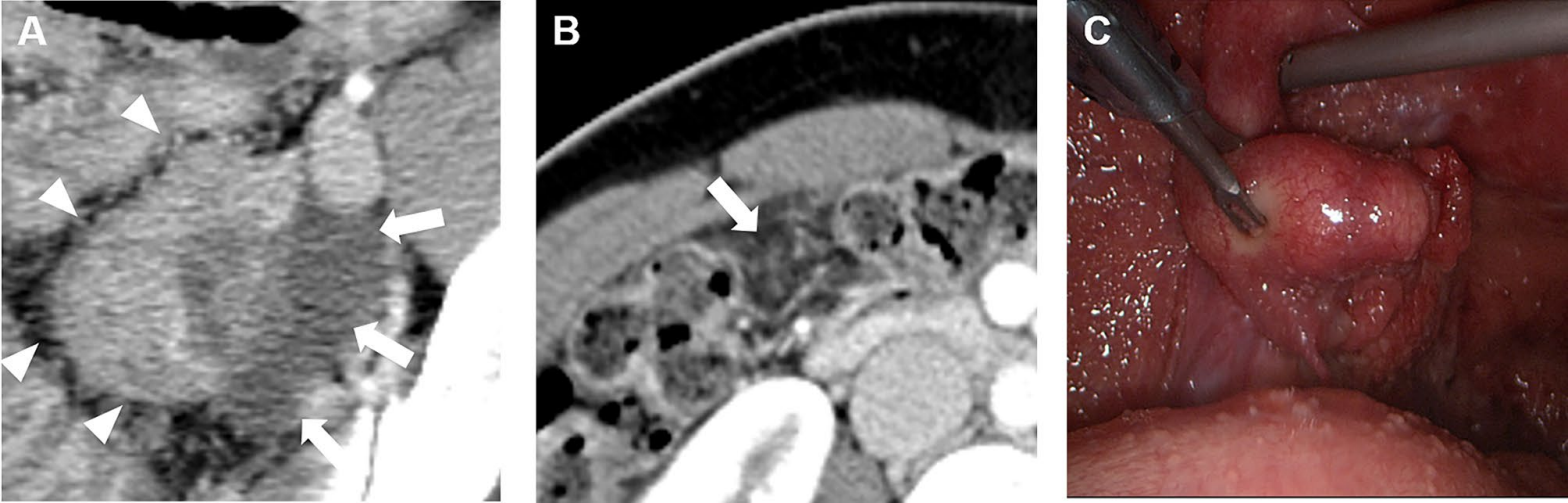
A case of TB peritonitis in a 28-year-old Korean woman. (**A**) In CT, there were bilateral ovarian masses [Rt. 4.9 cm (not shown), Lt. 4.5 cm (arrowheads)] in the pelvic cavity with hydrosalpinx (arrows). (**B**) There were diffuse peritoneal infiltration and nodularity without discrete peritoneal nodules or masses > 5 mm, considered peritoneal micronodules (arrow). The levels of CA125 and CA19-9 were 104.4 IU/mL and 1.3 IU/mL, respectively. Laparoscopic bilateral salpingectomy and left ovarian cystectomy were performed. (**C**) Diagnostic laparoscopy showed multiple miliary nodules on the uterine and peritoneal surfaces.

Table 2 shows the characteristics of CT imaging features of TB peritonitis and peritoneal carcinomatosis. Women with TB peritonitis had more changes in their fallopian tube, more peritoneal micronodules, and fewer macronodules than women with peritoneal carcinomatosis (64.0% vs. 8.8%, *p* < 0.001; 52.0% vs. 23.5% and 48.0% vs. 76.5%, *p* = 0.024). In the univariate logistic regression analysis using significantly different imaging features between the two diseases, changes in fallopian tubes, no adnexal mass, and peritoneal micronodules were significantly associated with TB peritonitis (*p* ≤ 0.027). In the subsequent multivariate analysis, changes in fallopian tube and peritoneal micronodules were independent parameters associated with TB peritonitis (*p* ≤ 0.012) (Table 3). When we combined the two independent imaging parameters, the AUC for differentiating TB peritonitis from peritoneal carcinomatosis was 0.855, with a sensitivity of 88.0% (95% confidence interval [CI], 68.8–97.5]. The specificity was 67.7% (95% CI 49.5–82.6), the positive predictive value was 66.7% (95% CI 54.6–76.9), the negative predictive value was 88.5% (95% CI 72.1–95.8), and diagnostic accuracy was 77.8% (95% CI 65.1–87.6). The kappa values for changes in fallopian tube and peritoneal micronodules were 0.708 and 0.778, respectively, which indicated a good agreement for the imaging features. A representative case of TB peritonitis in a CT scan and diagnostic laparoscopy is shown in Fig. 3.

**Table 2:** Discriminant CT imaging features between TB peritonitis and peritoneal carcinomatosis.

Imaging features	TB peritonitis (n = 25)	Peritoneal carcinomatosis (n = 34)	*P-*value
Changes in fallopian tubes			
Absent	9 (36.0)	31 (91.2)	< 0.001
Present	16 (64.0)	3 (8.8)	
Adnexal mass			
Absent	6 (24.0)	0	0.002
Present	19 (76.0)	34 (100)	
Mean ovary attenuation (HU)*	63.4 (33.8–95.7)	73.3 (47.0–95.0)	0.078
Peritoneal nodule			
Micronodules	11 (44.0)	2 (5.9)	0.024
Macronodules	14 (56.0)	32 (94.1)	
Omental pattern			
None	0	1 (2.9)	0.693
Infiltration	2 (11.1)	5 (14.7)	
Nodular	7 (25.9)	9 (26.5)	
Caked	16 (63.0)	19 (55.9)	
Ascites			
None	0	2 (5.9)	0.531
Small	5 (20.0)	6 (17.6)	
Moderate	5 (20.0)	4 (11.8)	
Large	15 (60.0)	22 (64.7)	
Mean ascites attenuation (HU)*	14.9 (5.4–24.3)	15.3 (7.2–33.1)	0.584

Data are the number of patients (percentage).

*HU* Hounsfield unit.

*Data are presented as the median (range) of the variables.

**Table 3:** Univariate and multivariate logistic regression analysis for differentiation of TB peritonitis from peritoneal carcinomatosis.

	Univariate analysis				Multivariate analysis		
	AUC	Exp (B)	95% CI	*P-*value	Exp (B)	95% CI	*P*-value
Changes in fallopian tubes	0.776	18.370	4.356–77.474	< 0.001	29.991	5.405–166.422	< 0.001
No adnexal mass	0.620			> 0.99			
Peritoneal micronodules	0.691	12.571	2.457–10.739	0.002	8.657	1.016–73.787	0.048

*AUC* the area under the receiver operating characteristics curve, *CI* confidence interval.

### Histopathologic pathologic results in comparison of two diseases

In histopathological analysis, all salpinges of peritoneal carcinomatosis of AEOC showed loss of cilia and the presence of atypia in the tubal epithelium. In contrast, the salpinges of TB peritonitis revealed dilated and congested blood vessels and fibrosis in the serosa surface in all five cases. Three cases of salpinges showed multiple epithelioid cell granulomas with multinucleated giant cells on the serosal surface. In addition, the epithelial cells lining the glands appear bland. Among the five patients, the CT scans for four patients showed changes in their fallopian tubes.

## Discussion

Women with TB peritonitis and peritoneal carcinomatosis of AEOC generally present at the hospital with abdominal distension or huge ascites. Pretreatment diagnosis if pivotal because the principles differ between the two diseases. Therefore, earlier studies focused on the differential diagnosis between TB peritonitis and peritoneal carcinomatosis of AEOC. However, most were either case reports or case series, and only a few original articles reported CT differential diagnoses of the two diseases. These studies were limited in that they included a small number of women with TB peritonitis and various types of primary malignancies as a comparative group, in addition to AEOC. Furthermore, their results are unlikely to be practical for actual image interpretation because ambiguous image findings are difficult to interpret^12,13^.

The following points are important for differentiating the two diseases in our study: first, if changes in fallopian tubes were observed in CT scan of women with abdominal distension or huge ascites, these changes showed an independent association with TB peritonitis rather than peritoneal carcinomatosis of AEOC. Because it is difficult to diagnose changes in fallopian tube at the serous tubal intraepithelial carcinoma (STIC) stage on both visual assessments in surgery and imaging studies, most STIC studies have focused on histopathologic findings. A lesion formerly referred to as primary fallopian tube carcinoma (PFTC) is on the STIC spectrum. Only one study reported that tubal cancer showed a sausage-shaped mass in the fallopian tube in a CT scan^16^. In contrast, the fallopian tube and endometrium are the most common sites for FGTB. Thickening and enhancement of salpinges with or without hydrosalpinx due to tubal obstruction are known imaging findings of TB salpingitis^17^. In our study, five patients underwent unilateral or bilateral adnexectomy. They all showed abnormalities on the serosal surface of the fallopian tube on histopathologic analysis, and four out of five patients showed fallopian tube abnormalities in CT. This finding is important because abnormalities in the fallopian tube, a characteristic region of FGTB, are correlated with histopathologic abnormalities.

Second, peritoneal micronodules were another characteristic CT imaging feature that differentiates TB peritonitis from peritoneal carcinomatosis. Peritoneal micronodules were more frequently observed in TB peritonitis than in peritoneal carcinomatosis (52.0% vs. 23.5%). In contrast, peritoneal macronodules were more frequently observed in peritoneal carcinomatosis than in TB peritonitis (76.5% vs. 48.0%). However, Ha et al. reported a higher frequency of peritoneal macronodules in TB peritonitis^13^. In contrast to our study, they included different comparison groups of peritoneal carcinomatosis, not only serous carcinoma but also other types of primary malignancies. In addition, they separated the omentum and mesentery abnormalities in CT scans, and the presence of micronodules or macronodules was evaluated only in the mesentery. However, it is often difficult to distinguish peritoneal nodules confined to specific anatomic locations because both TB peritonitis and peritoneal carcinomatosis present peritoneal nodules of various sizes and locations in the diffuse peritoneal disease. Thus, our image analysis defined macronodules when any nodules in the peritoneal cavity ≥ 5 mm in diameter. We defined the remaining cases as micronodules, which facilitated practical image interpretation. Given that the miliary nodule is a well-known laparoscopic and histopathologic finding of TB peritonitis, peritoneal nodules appear as micronodules rather than macronodules in CT scans. The combination of the changes in fallopian tube and peritoneal micronodules showed good diagnostic performance for differentiating TB peritonitis from peritoneal carcinomatosis of AEOC.

The differential diagnosis between TB peritonitis and peritoneal carcinomatosis has been challenging for a long time. Some studies used various imaging modalities to differentiate TB peritonitis from peritoneal carcinomatosis. CT was the most frequently studied modality for differentiating TB peritonitis from peritoneal carcinomatosis, with a sensitivity of 74.6–88.4% and specificity of 78.2–97.0%^18,19^. In addition, there was an attempt to differentiate peritoneal carcinomatosis from TB peritonitis using ultrasonographic omental thickness with a sensitivity of 89.3% and specificity of 84.1%^20^. Another previous study with fluorodeoxyglucose (FDG)-positron emission tomography (PET)-CT tried to diagnose TB peritonitis based on differential findings of two diseases, with a range of sensitivity of 60.0–80.0% and specificity of 80.4–94.1%^21^. Compared with our study, however, those previous studies included male and female patients and did not unify the cause of peritoneal carcinomatosis.

In contrast, we focused on female patients and peritoneal carcinomatosis by small-sized ovarian cancer (< 4 cm) because it is more challenging to differentiate TB peritonitis when there is a small-sized ovarian cancer mass and diffuse peritoneal disease^12^. In addition, because fertility preservation is pivotal in female patients of reproductive age, the differential diagnosis of TB peritonitis from peritoneal carcinomatosis of AEOC gives a chance to avoid radical and unnecessary surgery. A few studies used MRI for differentiating TB peritonitis from peritoneal carcinomatosis. MRI has a better soft tissue resolution than CT: therefore, MRI might help characterize adnexal abnormality in both diseases. This aspect of tumor imaging requires further research.

Our study had several limitations. The first limitation is the retrospective nature of our study. Second, the numbers of patients with TB peritonitis and peritoneal carcinomatosis of AEOC were still small.

In conclusion, changes in fallopian tube and peritoneal micronodules in pretreatment CT scans can differentiate TB peritonitis from peritoneal carcinomatosis of AEOC, which allows proper patient transfer to an expert gynecologist while avoiding unnecessary surgical procedures.

## Data Availability

The datasets generated and/or analyzed during the current study are not publicly available due to personal information protection but are available from the corresponding author upon reasonable request.

## Abbreviations

AEOC: Advanced epithelial ovarian cancer
AUC: Area under the receiver operating characteristics curve
CI: Confidence interval
CT: Computed tomography
FGTB: Female genital tuberculosis
HU: Hounsfield unit

## References

[1] WHO. WHO. WHO Global Tuberculosis report 2020. WHO https://apps.who.int/iris/bitstream/handle/10665/336069/9789240013131-eng.pdf. Accessed Apr 24, 2021. (2020).

[2] Muneer A, Macrae B, Krishnamoorthy S, Zumla A (2019). Urogenital tuberculosis—epidemiology, pathogenesis and clinical features. Nat. Rev. Urol..

[3] Altez-Fernandez C, Seas C, Zegarra L, Ugarte-Gil C (2016). Diseases masking and delaying the diagnosis of urogenital tuberculosis. Ther. Adv. Urol..

[4] Menzies NA (2018). Progression from latent infection to active disease in dynamic tuberculosis transmission models: A systematic review of the validity of modelling assumptions. Lancet Infect. Dis..

[5] Das P, Ahuja A, Gupta SD (2008). Incidence, etiopathogenesis and pathological aspects of genitourinary tuberculosis in India: A journey revisited. Indian J. Urol..

[6] Wise GJ, Marella VK (2003). Genitourinary manifestations of tuberculosis. Urol. Clin. N. Am..

[7] O'Flynn D (1970). Surgical treatment of genito-urinary tuberculosis. A report on 762 cases. Br. J. Urol..

[8] Koc S (2006). Peritoneal tuberculosis mimicking advanced ovarian cancer: A retrospective review of 22 cases. Gynecol. Oncol..

[9] Vázquez Muñoz E, Gómez-Cerezo J, Atienza Saura M, Vázquez Rodriguez JJ (2004). Computed tomography findings of peritoneal tuberculosis. Clin. Imaging.

[10] Aslan B, Tuney D, Almoabid ZAN, Ercetin Y, Seven IE (2019). Tuberculous peritonitis mimicking carcinomatosis peritonei: CT findings and histopathologic correlation. Radiol. Case Rep..

[11] Fahmi MN, Harti AP (2019). A diagnostic approach for differentiating abdominal tuberculosis from ovarian malignancy: A case series and literature review. BMC Proc..

[12] Shim SW, Shin SH, Kwon WJ, Jeong YK, Lee JH (2017). CT Differentiation of female peritoneal tuberculosis and peritoneal carcinomatosis from normal-sized ovarian cancer. J. Comput. Assist. Tomogr..

[13] Ha HK (1996). CT differentiation of tuberculous peritonitis and peritoneal carcinomatosis. AJR Am. J. Roentgenol..

[14] Oge T (2012). Peritoneal tuberculosis mimicking ovarian cancer. Eur. J. Obstet. Gynecol. Reprod. Biol..

[15] Wu CH (2011). Disseminated peritoneal tuberculosis simulating advanced ovarian cancer: A retrospective study of 17 cases. Taiwan J. Obstet. Gynecol..

[16] Ma FH (2015). MRI for differentiating primary fallopian tube carcinoma from epithelial ovarian cancer. J. Magn Reson. Imaging.

[17] Rezvani M, Shaaban AM (2011). Fallopian tube disease in the nonpregnant patient. Radiographics.

[18] Sohail AH (2022). Diagnostic accuracy of computed tomography in differentiating peritoneal tuberculosis from peritoneal carcinomatosis. Clin. Imaging.

[19] George S, Sathyakumar K, Bindra MS, Eapen A (2022). Is MDCT an accurate tool to differentiate between benign and malignant etiology in diffuse peritoneal disease?. Abdom. Radiol. (NY).

[20] Salman MA (2020). Predictive value of omental thickness on ultrasonography for diagnosis of unexplained ascites, an Egyptian centre study. Asian J. Surg..

[21] Wang SB (2017). PET/CT for differentiating between tuberculous peritonitis and peritoneal carcinomatosis: The parietal peritoneum. Medicine.

